# Physiological Profiles of Male Adolescents in Kelantan, Malaysia Practicing Taekwondo and Wushu

**DOI:** 10.21315/mjms2024.31.4.14

**Published:** 2024-08-27

**Authors:** Nur Fadhilah Ain Adanan, Foong Kiew Ooi, Norsuriani Samsudin

**Affiliations:** 1 Exercise and Sports Science Programme, School of Health Sciences, Universiti Sains Malaysia, Kelantan, Malaysia; 2 Faculty of Hospitality, Tourism and Wellness, Universiti Malaysia Kelantan, Kelantan, Malaysia

**Keywords:** anaerobic capacity, muscular strength, physiological profile

## Abstract

**Background:**

Martial arts training is beneficial for improving physical fitness but the improvements can vary according to the type of martial art performed. This study investigated lung function, aerobic and anaerobic capacity, flexibility, muscular strength and power among male adolescents who were sedentary or who practiced taekwondo or wushu.

**Methods:**

A total of 30 male adolescents living in the Kelantan state in Malaysia who were between 14 years old and 20 years old were enrolled and divided into three groups: i) sedentary (control), ii) taekwondo and iii) wushu. Each participant underwent a lung function test, 20 m shuttle run, Wingate anaerobic test and a Sit and Reach test, as well as tests of standing long jump power, handgrip, back and leg strength.

**Results:**

Relative to the sedentary control group, the wushu group had significantly higher values than the sedentary control group for fat free mass (*P* = 0.047), explosive leg power (*P* < 0.001), aerobic capacity (*P* < 0.001), forced expiratory volume in 1 s (FEV_1_) (*P* = 0.021) and Wingate anaerobic capacity (*P* < 0.05). The taekwondo group also showed significantly greater values than the sedentary control group for explosive leg power (*P* = 0.018), forced vital capacity (FVC) (*P* = 0.014) and FEV_1_ (*P* < 0.001). The wushu group exhibited significantly higher explosive leg power (*P* = 0.010) and Wingate anaerobic capacity parameters including mean power (*P* = 0.001), anaerobic capacity (*P* < 0.001) and anaerobic power (*P* = 0.002) than the taekwondo group.

**Conclusion:**

Engagement of male adolescents in wushu and taekwondo was associated with improved physiological profiles compared to those who were in the sedentary control group. Practice of wushu could result in greater explosive leg power and anaerobic capacities than taekwondo.

## Introduction

Martial arts are defined as offensive and defensive combat systems that are modified for modern sports and exercise. Moreover, the practice of martial arts involving unarmed training and/or practice with weapons can play a role in achieving spiritual growth (1). The term ‘martial arts’ is often broadly applied for different disciplines, which include karate, judo, soo bahk do, taekwondo, wushu and tai chi. Many studies showed that practicing martial arts provides various physiological benefits, such as increases in muscular strength, anaerobic capacity, balance, and flexibility, as well as an overall improvement in cardiorespiratory fitness (2).

Taekwondo is a martial art that originated in Korea and is characterised by fast, high and spinning kicks. The word ‘taekwondo’ refers to the art of kicking and punching and the roots of the word, ‘tae’, ‘kwon’ and ‘do’, correspond to ‘hit using the foot’, ‘hit using the fist’ and ‘art’, respectively. Taekwondo is unique in its predominant use of powerful kicking techniques (3). Traditional and modern styles of Chinese martial arts have different sets of techniques and ideas. Wushu, which is also known as ‘kung fu’, is one type of millenary martial art that originated in China (4). Various forms of Chinese culture, such as philosophy, art, literature, religion and ethics have influenced wushu (5), which now involves a variety of offensive and defensive fighting techniques that can be divided into numerous styles and schools.

Martial arts require comprehensive motor fitness that includes muscle strength, endurance and speed. By performing combinations of movements with high intensity in short bursts, practitioners of martial arts develop above-average coordination and flexibility while integrating strength with velocity and endurance that impact athletes’ overall physical development (6–8). Martial arts require maximum muscle power and endurance, particularly in the lower limbs that are needed to execute fast and powerful movements (3). A recent study by Stamenković et al. (9) showed that practicing martial arts leads to enhanced physical fitness as manifested by increased cardiorespiratory fitness, speed, agility, strength, flexibility, coordination and balance.

Maximal oxygen uptake (VO_2max_) reflects the delivery of oxygen from the cardiovascular system to working muscles. Individuals who have high VO_2max_ values are traditionally regarded as possessing ‘endurance fitness’ or ‘cardiorespiratory fitness’. Meanwhile, anaerobic capacity is important for short bursts of high-intensity activity, when the body’s demand for oxygen exceeds available oxygen supplies and energy sources stored in muscles are used (10). Kabadayi et al. (11) studied physical fitness of karate practitioners and found that these individuals had high levels of cardiorespiratory and muscular endurance, as well as anaerobic capacity. In another study that measured other physiological parameters to compare taekwondo and karate athletes, Gunawan (12) reported that taekwondo athletes had greater flexibility than karate athletes, whereas karate athletes had greater leg strength than taekwondo athletes. In addition, Andreato et al. (13) found that elite Brazilian Jiu-Jitsu athletes had medium flexibility and excellent maximal isometric back strength.

Measurements of physiological profiles of individuals provide important information for the selection of potential athletes, as well as the design and develop training programmes to enhance sports performance. We carried out several recent related studies that compared physical fitness components or physiological profiles among athletes engaged in different sports such as boxing, Muay Thai and silat (14), weightlifting, cycling and squash (15), as well as rugby and hockey (16).

Taekwondo and wushu are types of martial arts that are commonly practiced by males but physiological profiles of individuals who engage in these activities in Malaysia are limited. The fitness components of taekwondo and wushu may differ between the two disciplines due to different physiological demands that each entail. In this study, we compared differences in lung function parameters, aerobic and anaerobic capacities, flexibility, muscular strength and power among three study groups of males: i) sedentary (control), ii) taekwondo and iii) wushu practitioners.

## Methods

### Study Settings and Sampling

For this cross-sectional study, 30 Malaysian male adolescents aged between 14 years old and 20 years old were assigned into three groups of 10: i) sedentary (control), ii) taekwondo and iii) wushu. The inclusion criteria for the taekwondo and wushu participants were individuals who had practiced taekwondo or wushu at least two times per week for at least 2 years. Inclusion criteria for sedentary participants were those who were not involved in any competitive sports and who exercised fewer than two times per week. Individuals who had any injury or acute and chronic diseases, as well as those who reported consistent intake of medication or nutrition supplements during the 6 months before the study begins were excluded. All participants who met the study eligibility criteria provided written consent.

This study used an opportunistic sampling method. Participants were recruited from the population of the Kelantan state in Malaysia. Members of the sedentary (control) group were recruited through teachers in a secondary school, whereas those in the taekwondo and wushu groups were recruited through taekwondo and wushu coaches. During the first meeting, potential participants were briefed on the study details, which included inclusion and exclusion criteria and potential risks. The participants were then screened to determine if the inclusion and exclusion criteria were met. Those who met the criteria and agreed to participate were required to complete the consent form. This research was conducted at the Sport Science Laboratory, Health Campus, Universiti Sains Malaysia.

### Sample Size Calculation

The sample size was calculated using GPower software. The power of the study was set at 80% with a 95% confident interval, i.e. alpha = 0.05 and the effect size was set at 0.60 for the three study groups. The total number of participants calculated was 30. Since three groups of participants were recruited, 10 participants were recruited for each group.

### Anthropometric Measurements, Flexibility, Handgrip, Back and Leg Strength and Explosive Power Test Using Standing Long Jump

Anthropometric parameters such as body weight (kg) and body height (cm) were measured with a stadiometer (Seca 220, Hamburg, Germany) while the participants were wearing lightweight clothing and no shoes. To assess flexibility of the lower back and hamstring muscles, participants performed a Sit and Reach test. A hand dynamometer (JAMAR J00105, USA) was used to measure the strength of the participants’ handgrip. A back and leg dynamometer (Takei TKK 1858, Japan) was used to measure participants’ back and leg strength. The explosive power of participants’ leg muscles was assessed using a standing long jump test.

### Measurement of Lung Function Parameters

Lung function parameters for the participants including forced vital capacity (FVC) and forced expiratory volume in 1 s (FEV_1_) were measured using a Bellow spirometer (Vitalograph, Buckingham, United Kingdom).

### Aerobic Capacity and Wingate Anaerobic Tests

The estimated oxygen uptake VO_2max_ of the participants was determined with a 20 m shuttle run test in which the participants ran 20 m in time with a ‘beep’ sound played from a CD player. The run was repeated until the participants could not keep pace with the ‘beep’. The estimated VO_2max_ was calculated based on the number of completed laps using a tool at the website (http://www.topendsport.com/testing/beepcalc.htm). In a Wingate anaerobic capacity test, the participants performed a 30-s maximal effort on a cycle ergometer (H-300-RLode, Groningen, Holland).

### Statistical Analysis

Statistical analyses were carried out using Statistical Package for Social Sciences (SPSS) version 22.0. The normality distribution of the data was checked. The data were confirmed to be normally distributed and thus, a one-way ANOVA followed by Bonferroni post hoc test was used to determine differences in means of the measured parameters among the groups. All data are presented as means and standard deviation (SD). The accepted level of significance was set at *P* < 0.05.

## Results

### Participant Anthropometry

A total of 30 male participants were assigned to three groups of 10 each: i) sedentary (control), ii) wushu and iii) taekwondo. All members of the sedentary group were Malays and all members of both the wushu and taekwondo groups were Malaysian Chinese. The mean age of all the participants was 15.6 (SD = 1.5) years old and the mean ages for the three groups were 15.6 (SD = 1.4) years old, 15.5 (SD = 2.0) years old and 15.6 (SD = 1.3) years old for the sedentary (control), wushu and taekwondo groups, respectively. The mean body height for the taekwondo group [170.4 (SD = 7.2) cm] was significantly higher (*P* = 0.004) than the sedentary (control) group [158.9 (SD = 7.3) cm], whereas the wushu group mean height [163.6 (SD = 7.2) cm] showed no significant difference from the taekwondo group or sedentary control group. The body weight for taekwondo group was also significantly higher (*P* = 0.13) than that for the sedentary (control) group [62.6 (SD = 16.9) kg] versus [44.9 (SD = 10.2) kg] but the wushu mean group body weight [53.8 (SD = 9.5) kg] showed no significant difference from the taekwondo or sedentary (control) groups.

### Flexibility, Hand Grip Strength, Back and Leg Strength and Standing Long Jump Explosive Power

**Figure 1:**
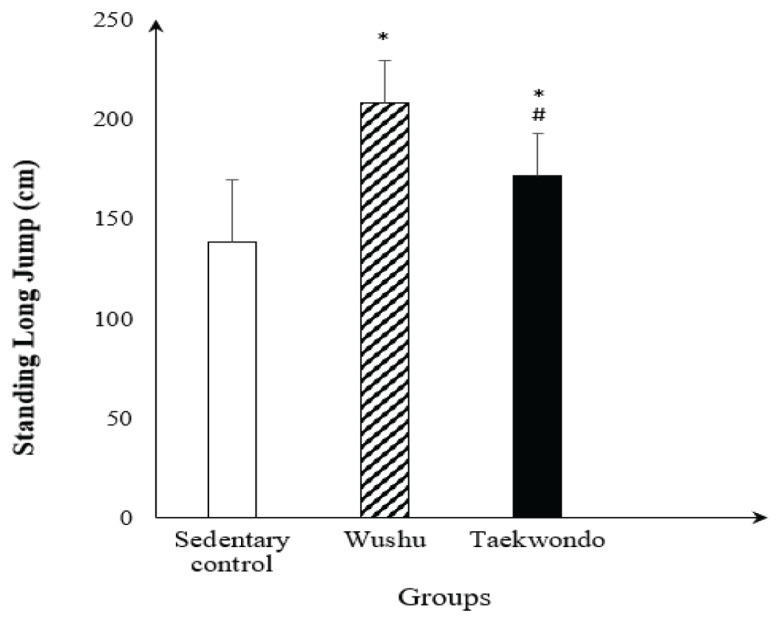
SLJ power in sedentary (control), wushu and taekwondo groups Notes: Values are expressed as mean (SD); *P < 0.05, statistically significant different from sedentary (control) group; ^#^*P* < 0.05, statistically significant different from wushu group

In the standing long jump power test (SLJ), the wushu and taekwondo groups showed significantly greater jumping distance (*P* < 0.001 and *P* = 0.018, respectively) than the sedentary (control) group; and the wushu group had significantly greater jumping distance (*P* = 0.010) than the taekwondo group (Figure 1). No significant differences in flexibility, hand grip strength and back and leg strength among the groups were observed (Table 1).

### Aerobic Capacity (Estimated VO_2 max_), Lung Function Parameters and Wingate Anaerobic Capacities

Relative to the sedentary (control) group, the wushu group showed significantly higher estimated VO_2max_ (*P* < 0.001) and FEV_1_ (*P* = 0.021), whereas the taekwondo group had significantly greater FVC (*P* = 0.014) and FEV_1_ (*P* = 0.001) (Table 2). The wushu and taekwondo groups had no significant differences in estimated VO_2 max_ and all measured lung function parameters (Table 2).

Wingate anaerobic capacities (ACs) measured for the sedentary (control), wushu and taekwondo groups indicated that the wushu group had significantly greater mean power (MP; *P* < 0.001), peak power (PP; *P* = 0.007) and AC (*P* < 0.001) compared to the sedentary control group (Table 3). The wushu group also exhibited significantly higher MP (*P* = 0.006), AC (*P* < 0.001) and anaerobic power (AP) (*P* = 0.002) compared to the taekwondo group.

## Discussion

Explosive power is an important element for the competitive success of martial arts athletes and refers to the ability of an athlete to generate quick movements with maximal effort. A combination of biomechanical parameters, including velocity force, acceleration and momentum contributes to explosive power and overall high athletic performance (17). In a study of youth involved in basketball and football, Pinero et al. (18) demonstrated that SLJ is strongly associated with muscular strength. In the present study, we found that study participants who had at least 2 years of twice-weekly training in wushu and taekwondo exhibited significantly greater SLJ explosive power compared to the sedentary (control) participants. These results indicated that both wushu and taekwondo training were effective in enhancing leg explosive power.

In this study, the wushu group showed significantly greater explosive jumping power compared to the taekwondo group. This difference may have been due to the techniques, skills and movements associated with wushu training that requires continuous changes in intensity and kinetic movements and is characterised by multiple lateral movements, jumps, throws and body contact, which are all highly dependent on muscular power. In a study of mechanical jumping power of male wushu players, Kumar et al. (19) showed that explosive power is an important element at the elite level of competitive wushu. This fitness component helps wushu practitioners execute both attack or defense techniques. Veligekas et al. (20) also reported that SLJ performance was significantly correlated with explosive leg power and lower body muscular strength. The findings of the present study are consistent with these earlier studies in terms of the finding that wushu practitioners have greater explosive leg power than taekwondo practitioners.

Meanwhile, we observed no significant differences in flexibility or hand grip, back and leg strength among the groups. Measurements were carried out for all participants to assess hamstring, low back and hip joint range of motion and flexibility, which is required not only for daily activities but also for effective athletic training, particularly in the martial arts. In this study, we saw no significance difference in flexibility when comparing the sedentary (control) group with the wushu and taekwondo groups or between the wushu and taekwondo groups. In contrast, a study by Artioli et al. (4) investigating the physiological and performance profiles of Olympic wushu athletes in Brazil found that all study participants had high values for flexibility. This contrasting result could be due to the differences in age between the participants, as our study had young athletes with less experience and training in wushu.

Handgrip strength for martial arts is critical (21, 22). In our study, we did not record significant differences in handgrip strength among the three study groups. Heller et al. (23) compared handgrip strength between male and female elite taekwondo athletes from the Czech national team and found that males had higher grip strength than females. Meanwhile, Andreato et al. (13) reported that the maximal isometric handgrip strength for Brazilian Jiu-jitsu athletes was lower than that for judo athletes, who must sustain greater force during matches that can be achieved by development of maximal isometric handgrip strength. Their results also showed lower values for handgrip strength than other studies concerning athletes in different grappling combat sports but was similar to results for junior wrestlers. The lack of significant differences in handgrip strength seen in the present study could again be due to the younger age of the study participants.

Back and leg strength is another important factor in martial arts success. Andreato et al. (13) reported that elite Brazilian Jiu-jitsu athletes had greater maximal isometric back strength than that reported in other studies involving judo athletes and junior athletes. Demirkan et al. (24) showed a significant difference in back strength for junior elite wrestlers compared to sedentary (controls). However, in our study, we found no significance differences in back and leg strength among the wushu, taekwondo and sedentary groups. This similarity could also be attributed to the relative youth and inexperience of the study participants.

Taekwondo is both aerobic and anaerobic and involves short bursts of rapid and high-intensity movements separated by lower-intensity movements (25). Systematic training in wushu or kung fu can also improve cardiovascular, aerobic and anaerobic fitness. In a study of the physiological profiles of members of the Iranian national wushu team, Sanchooli et al. (26) found that wushu athletes have high AP in their lower limbs. Tsang et al. (27) found that kung fu training with moderate-to-high intensities ranging from 52.4% to 82.1% of VO_2max_ are adequate for eliciting cardiovascular benefits.

In our study, the wushu group showed significantly greater estimated VO_2max_ relative to the sedentary (control) group. Participants in the wushu and taekwondo groups also exhibited higher values in lung function parameters compared to the sedentary (control) group. Meanwhile, the estimated VO_2max_ and lung function parameters were similar between the wushu group and taekwondo groups, suggesting that practice of either wushu or taekwondo could enhance aerobic fitness and lung functions compared to a sedentary lifestyle.

For aerobic fitness, Heller et al. (23) showed that male taekwondo athletes had better AC than the general population. In an investigation of the physiological profile of recreational taekwondo practitioners, Toskovic et al. (28) found that those who attained black belt qualifications and had at least 3 years of training had better aerobic power than novice practitioners who had only 8 weeks of training. Fong and Ng (25) compared the cardio-respiratory profile of taekwondo participants with age-matched sedentary population and ultra-distance runners. They found that taekwondo practitioners had poorer cardio-respiratory fitness than ultra-distance runners, reflecting the lower emphasis of taekwondo training on the development of aerobic fitness compared to ultra-distance running.

Another notable finding in our study is that the wushu groups showed significantly greater AC parameters, i.e. MP, PP and AC compared to the sedentary (control) group. The wushu group also showed significantly greater MP, AC and AP compared to the taekwondo group. These findings implied that wushu training could elicit higher ACs than taekwondo training and the sedentary (controls). This difference could be due to the importance of short bursts of energy needed for wushu rather than continuous effort. Such short bursts require high AC.

This study has several limitations. First, the participants were relatively young, between 14 years old and 20 years old. Second, the ethnicity of participants in both the taekwondo and wushu groups was Chinese, while all the participants in the sedentary (control group) were Malays. Third, the participants competed at the state level and thus were comparatively inexperienced. Last, the study sample size was small. Together, these limitations could restrict the generalisability and statistical power of the study findings. Future studies involving a larger sample size having a greater range of age and ethnicities are needed, as is the inclusion of athletes having different levels of experience. Furthermore, studies to compare taekwondo and wushu with other types of martial arts such as judo, karate, wrestling, Muay Thai and boxing are needed.

## Conclusion

This study found that adolescent male wushu practitioners have greater explosive leg power and ACs than taekwondo practitioners. Explosive leg power and ACs are two essential physical components of wushu. We found that training in wushu could have positive effects on fat free mass, explosive leg power, aerobic capacity, lung function and AC compared to a sedentary lifestyle. Meanwhile, engagement in taekwondo could enhance fat free mass, explosive leg power and lung functions compared to a sedentary lifestyle. The findings from this study can be used as guidelines for coaches of taekwondo and wushu to design training programmes that focus on improving essential physical fitness components. Our results can also be used as guidelines to promote active lifestyles that involve training in martial arts like taekwondo and wushu.

## Figures and Tables

**Table 1:** Flexibility, handgrip strength and back and leg strength in sedentary control, wushu and taekwondo groups

	Sedentary (control) group (*n* = 10)	Wushu group (*n* = 10)	Taekwondo group (*n* = 10)	Difference in mean and 95% Confidence interval		

				Sedentary control versus wushu	Sedentary control versus taekwondo	Wushu versus taekwondo
Flexibility (cm)	30.7 (8.9)	21.93 (5.1)	35.7 (14.5)	8.75 (−2.98, 20.49)	−5.03 (−16.77, 6.70)	−13.79 (−25.52, −2.05)
Hand grip strength (kg)						
Dominant hand	30.0 (10.0)	34.2 (4.5)	34.5 (8.8)	−4.24 (−13.53, 5.05)	−4.53 (−13.82, 4.76)	−0.30 (−9.58, 8.99)
Non-dominant hand	27.2 (10.0)	31.6 (5.2)	32.0 (7.0)	−4.40 (−12.90, 4.12)	−4.77 (−13.27, 3.74)	−0.38 (−8.88, 8.13)
Back and leg strength (kg)	91.5 (34.7)	108.6 (30.0)	120.2 (19.1)	−17.16 (−49.94, 15.61)	−28.73 (−61.51, 4.05)	−11.57 (−44.35, 21.21)

Note: Values are expressed as mean (SD)

**Table 2:** Aerobic capacity (estimated VO_2max_) and lung function parameters in sedentary (control), wushu and taekwondo groups

	Sedentary control group (*n* = 10)	Wushu group (*n* = 10)	Taekwondo group (*n* = 10)	Difference in mean and 95% Confidence interval		

				Sedentary (control) versus wushu	Sedentary (control) versus taekwondo	Wushu versus taekwondo
Estimated VO_2max_ (mL.kg.min^−1^)	28.8 (2.8)	**35.6 (2.7)** *	32.2 (4.2)	−6.73 (−10.49, −2.98)	−3.37 (−7.13, 0.39)	3.36 (−0.40, 7.12)
FVC (L)	2.8 (0.8)	3.6 (0.8)	**3.9 (0.8)** *	−0.81 (−1.70, 0.09)	−1.08 (−1.98, −0.18)	−0.28 (−1.17, 0.62)
FEV_1_ (L)	2.3 (0.7)	**3.2 (0.7)** *	**3.6 (0.7)** **	−0.88 (−1.64, −0.11)	−1.28 (−2.04, −0.51)	−0.40 (−1.17, 0.37)
FEV_1_/FVC ratio (%)	84.0 (18.6)	88.5 (6.1)	92.5 (4.4)	−4.56 (−17.81, 8.68)	−8.56 (−21.81, 4.68)	−4.0 (−17.24, 9.24)

Note: Values are expressed as mean (SD);

* *P* < 0.05,

** *P* < 0.01 = statistically significant different from sedentary control group;

VO_2max_ = maximal oxygen consumption; FVC = forced vital capacity; FEV_1_ = forced expiratory volume in 1 s

**Table 3:** Results of Wingate AC in sedentary (control), wushu and taekwondo groups

	Sedentary control group (*n* = 10)	Wushu group (*n* = 10)	Taekwondo group (*n* = 10)	Difference in mean and 95% Confidence interval		

				Sedentary (control) versus wushu	Sedentary (control) versus taekwondo	Wushu versus taekwondo
MP (Watt)	266.3 (79.6)	**472.3 (77.9)** *	**335.6 (108.7)** ##	−205.93 (−308.49, −103.37)	−69.23 (−171.79, 33.33)	136.7 (34.14, 239.26)
PP (Watt)	433.0 (65.8)	**606.8 (114.0)** **	514.9 (151.2)	−173.80 (−305.95, −41.65)	−81.90 (−214.05, 50.25)	91.90 (−40.25, 224.05)
AC (Watt.kg^−1^)	5.8 (1.3)	**8.8 (1.0)** *	**5.4 (1.3)** #	−3.04 (−4.42, −1.66)	0.38 (−1.01, 1.76)	3.42 (2.04, 4.80)
AP (Watt.kg^−1^)	9.6 (1.1)	**11.4 (2.0)**	**8.4 (2.1)** ##	−1.81 (−3.80, 0.18)	1.14 (−0.81, 3.13)	2.95 (0.96, 4.94)
FI (Watt.sec^−1^)	11.9 (4.1)	13.1 (4.4)	14.7 (8.1)	−1.24 (−7.87, 5.39)	−2.85 (−9.48, 3.78)	−1.61 (−8.24, 5.02)

Notes: Values are expressed as mean (SD);

* *P* < 0.05,

** *P* < 0.01 = statistically significant different from sedentary control group;

# *P* < 0.05,

## *P* < 0.01 = statistically significant different from wushu group;

MP = mean power; PP = peak power; AC = anaerobic capacity; AP = anaerobic power; FI = fatigue index
